# How to design real-world functional near-infrared spectroscopy studies: a primer

**DOI:** 10.1117/1.NPh.13.S1.S10701

**Published:** 2025-11-14

**Authors:** Isla L. Jones, Chiara Bulgarelli, Sara De Felice, Paola Pinti, Antonia F. de C. Hamilton

**Affiliations:** a University College London, Institute of Cognitive Neuroscience, London, United Kingdom; b Birkbeck, University of London, London, United Kingdom; c University of Cambridge, Department of Psychology, Cambridge, United Kingdom

**Keywords:** fNIRS, design, experiment, naturalistic, functional near-infrared spectroscopy, social, developmental, hyperscanning

## Abstract

**Significance:**

Functional near-infrared spectroscopy (fNIRS) is a unique neuroimaging methodology with high portability and tolerance of motion, making it well-suited to research in dynamic real-world environments. However, it is crucial to carefully design fNIRS paradigms to suit the unique requirements of real-world research settings. We outline key design principles and considerations for fNIRS studies.

**Aim:**

In this paper, we address the lack of guidance on experimental design for fNIRS, which in our growing field can help to educate new fNIRS researchers and improve the quality and applicability of fNIRS research across various settings.

**Approach:**

Here, we provide a primer for how to design fNIRS studies and overcome challenges in fNIRS experimental design, with a focus on naturalistic real-world research, which has gathered increased research interest in recent years.

**Conclusions:**

We conclude by outlining seven key design principles researchers can use to guide experimental design for fNIRS research.

## 1 Introduction

Functional near-infrared spectroscopy (or fNIRS) is a rapidly growing method with enormous potential for real-world neuroscience and social neuroscience. However, there is a significant challenge in understanding how best to design research studies using fNIRS in naturalistic contexts to effectively leverage this technology in nonconventional, relatively unconstrained settings. The question of how studies can be designed both to capture meaningful real-world behaviors and to make use of the massive flexibility of fNIRS devices is a major focus of this paper. This paper is a primer aimed at researchers with little or no expertise in experimental design. It sets out the core principles for designing tasks for cognitive neuroscience studies using fNIRS. First, we will introduce fNIRS and explore its strengths and limitations in Sec. 1.1. We will then go on to discuss general design principles for fNIRS research (Sec. 2), before specifically addressing naturalistic research (Sec. 3), hyperscanning research (Sec. 4), and developmental research (Sec. 5). We will conclude by reiterating key challenges and recommendations for fNIRS experimental design (especially in real-word settings), as well as highlighting potential future directions for the field.

### 1.1 What fNIRS Can and Cannot Do

fNIRS is an optical neuroimaging technology that uses near-infrared light to estimate relative changes in the concentration of oxygenated (HbO) and deoxygenated hemoglobin (HbR) in the blood.^1^ Based on neurovascular coupling,^2^ changes in HbO and HbR are considered indirect indicators of brain activity. This is because an increase in cerebral blood flow, alongside an increase in HbO and a decrease in HbR, is reliably observed following neuronal firing—this is known as the hemodynamic response. The hemodynamic response measured with fNIRS is directly comparable to the BOLD response measured in functional magnetic resonance imaging (fMRI), which leverages the paramagnetic properties of deoxygenated hemoglobin.^3^ However, fNIRS measures relative concentration changes in both HbO and HbR, and analyzing both signals can provide insight into physiological processes and aid interpretation. For example, Cui et al.^4^ developed an “activation signal” based on the anticorrelation between HbO and HbR, which likely reflects functional activation. Similarly, Yamada et al.^5^ developed a method to separate components of the fNIRS signal into the functional component (where HbO and HbR are negatively correlated) and the component affected by systemic physiology (which can lead to positive correlations between HbO and HbR). Further discussion of components of the fNIRS signal and how they may lead to false positives and false negatives can be found in previous work.^6^^,^^7^

fMRI can capture data from the whole brain (3 mm resolution and a frequency of 0.5 to 0.3 Hz is typical for whole brain recordings), whereas fNIRS captures data only from the cortical surface with no sensitivity to regions deeper than ∼2  cm. Most current fNIRS devices capture data at ∼25 to 30 mm resolution and 5 to 10 Hz, but newer diffuse optical tomography increases this to 6 mm resolution with the same frequency.^8^

Because fNIRS and fMRI are measuring the same brain signal, this means that fNIRS studies can draw on the principles of fMRI experimental design that have successfully been developed over decades of research. Principles of experimental design drawn from fMRI provide an excellent starting point for a new researcher learning to use fNIRS, and we summarize these in Sec. 2.1. However, fNIRS can also go beyond fMRI as it facilitates data collection in more complex contexts outside the classical and strictly controlled laboratory setting,^9^ thus widening the possibilities for different experimental designs, research questions, and related applications. Alternative neuroimaging modalities do not have such a wide range of uses. fMRI requires participants to remain completely still, allowing only limited hand and eye movements, and electroencephalography (EEG) recordings are highly susceptible to motion artifacts from even small eye or face movements. This makes these other modalities challenging for use outside the laboratory. Moreover, the higher temporal resolution of fNIRS compared with fMRI enables insights into the more rapid changes in the hemodynamic signal. This allows clearer measurement and tracking of physiological components of the signal, such as heartbeats, thus making it easier to separate these from the signal or extract these physiological signals for further processing. Therefore, higher temporal resolution facilitates more precise filtering and removal of artifacts in the fNIRS signal while avoiding aliasing of faster physiological components.

In contrast to fMRI, a key advantage of fNIRS is its tolerance to motion and portability, allowing it to be employed in real-world settings—especially the new generation of wireless and wearable devices.^10^ For example, researchers have used fNIRS to explore questions relating to dance,^11^^–^^13^ live theatre,^14^ and walking around a real-world street environment.^15^ fNIRS is also well suited to studies of social interactions. Participants wearing an fNIRS cap can take part in conversations,^16^^,^^17^ imitation tasks,^18^ cooperative tasks,^19^^,^^20^ and many other social activities, without drastically impacting signal quality. This flexibility is opening new research avenues in the domain of real-world social neuroscience and second-person neuroscience^21^^,^^22^ and enables studies of truly naturalistic social interactions.

However, the freedom to move outside the lab with fNIRS also brings new challenges in experimental design. Real-world behaviors and natural social interactions do not always fall into neat experimental blocks, with 30 s of one task followed by exactly 30 s of another, or with clean repeatable experimental conditions that can be averaged together. A key focus of this paper is how to design research studies that both capture meaningful real-world behaviors and take full advantage of the flexibility offered by fNIRS devices.

## 2 How Should We Design an fNIRS Study?

Selecting an appropriate experimental design is essential to the success of a neuroimaging research project, but this skill is rarely explicitly taught or described in textbooks. A good design should capture brain activity that is related to the function of interest, should be feasible to interpret without confounds, and should have a good signal-to-noise ratio in the data to enable appropriate statistical analysis. In this section, we will first give a general overview of fMRI relevant to fNIRS, followed by a discussion of the importance of a well-selected control condition.

### 2.1 Lessons from fMRI

The challenge of inferring cognitive processes from brain activity patterns is not new to the domain of fNIRS research. Researchers using fMRI (and before that, positron emission tomography) have developed core principles and sophisticated experimental designs over 30 years, to be able to link blood flow patterns to cognition. Here, we briefly review these principles, drawing on the work of Friston et al.^23^ and others.^24^^,^^25^

Most cognitive neuroimaging research begins with an (implicit) model of human cognition as a type of information processing, in which sensory information comes into the brain, is processed in a variety of ways and a motor response is emitted (see Fig. 2). This processing could involve memory, language, imitation, theory of mind, or any number of other cognitive systems depending on the task that is given to the participants. Although this type of “box and arrow” model has been extensively criticized, the basic idea that different regions of the brain contribute to different cognitive processes is still a core assumption in most cognitive neuroscience research. For a researcher planning a study, it is therefore very helpful to begin with an idea about what core cognitive process they wish to measure and how that process might change in different experimental conditions.

**Fig. 1:**
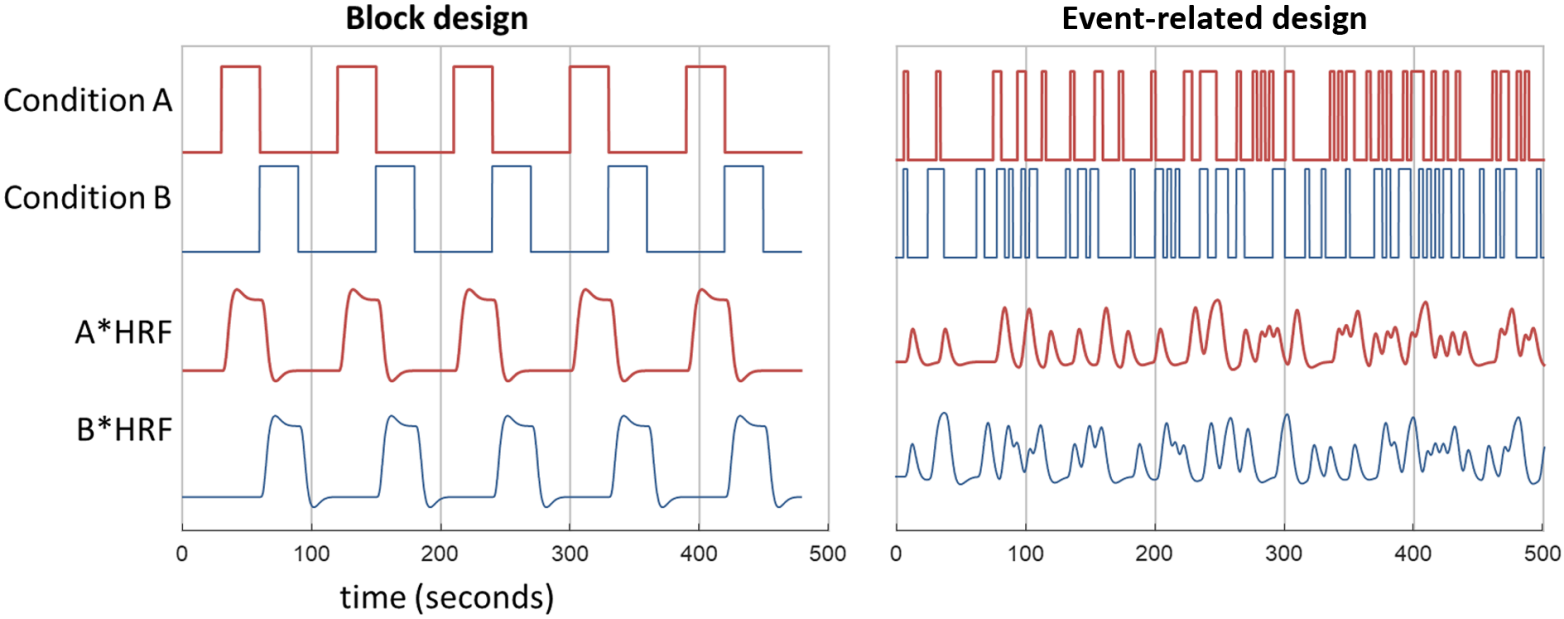
Classic designs for functional neuroimaging. In a block design, two conditions with long block durations lead to distinct predicted brain signals when convolved with an HRF. In an event-related design, the two conditions have short durations and do not alternate regularly but have pseudorandomized timing. This means that two distinct predicted brain signals can still be obtained when the short events are convolved with the HRF. Many different combinations and variations on these basic designs are also possible.

We begin by considering a classic experimental design comparing brain responses to condition A and condition B. For example, condition A might involve detecting images of faces, whereas condition B involves detecting images of houses.^26^ Typically, participants in such studies will see and respond to stimuli on a computer with the timing of the stimuli and task controlled by the experimenter, but what task timing and ordering should be used? The hemodynamic response function (or HRF) provides a convenient mathematical model of how neural activity relates to subsequent changes in HbO and HbR in that region (see Lindquist and colleagues^27^), and this response typically peaks ∼5  s after neural activity begins. Given this slow rise-time of the hemodynamic response, simulations show that alternating blocks of 30 s Task A/30 s Task B maximizes the power of the signal that can be recorded with fMRI or fNIRS.^24^ The left panel of Fig. 1 illustrates this classic block design, showing how the two convolved responses are distinct and separable. This classic block design has been widely used with both adults^26^ and infants.^28^

In all these examples, we are assuming that data will be analyzed using a general linear model approach (GLM)^23^ as used in much fMRI research. In this approach, the aim is to build a complete design matrix that captures all the tasks and events that matter in an experimental session, and then the fNIRS data from each channel are fit to the design matrix to provide a statistical test of which task/event best accounts for the data in that channel. In the example above, the design matrix would model the potential neural responses to faces and the potential neural responses to houses (A*HRF and B*HRF in Fig. 1). A design matrix can also include factors to model participant responses in a task (e.g., button press) or physiological variables (e.g., heart rate), allowing an integrative model of many aspects of a task. This contrasts with block-average approaches to fNIRS data, where distinct blocks of the task are separated from each other and then averaged.

The GLM approach is flexible because it can also deal with contexts where it is not plausible to separate stimuli or tasks into distinct 30 s blocks. An event-related design with much shorter events can still be used if the events have irregular timing and/or sequencing. This irregularity ensures that, after convolving the predicted brain signals with the hemodynamic response function, the signals from different events can be distinguished from one another. That is, the signals A*HRF and B*HRF in the right panel of Fig. 1 must not correlate with each other. If the two signals are too similar, they cannot be included as independent predictors in a general linear model (or design matrix).^29^ Using a GLM to analyze neuroimaging data allows the researcher to determine how well brain activity aligns with patterns expected for a given condition, based on the condition timings convolved with an HRF. If the events in tasks A and B occur too close together in time or follow a regular pattern, A*HRF and B*HRF will likely overlap and become too similar (colinear), making it impossible to separate them in the general linear model. This would result in unreliable estimates of brain activity.

**Fig. 2:**
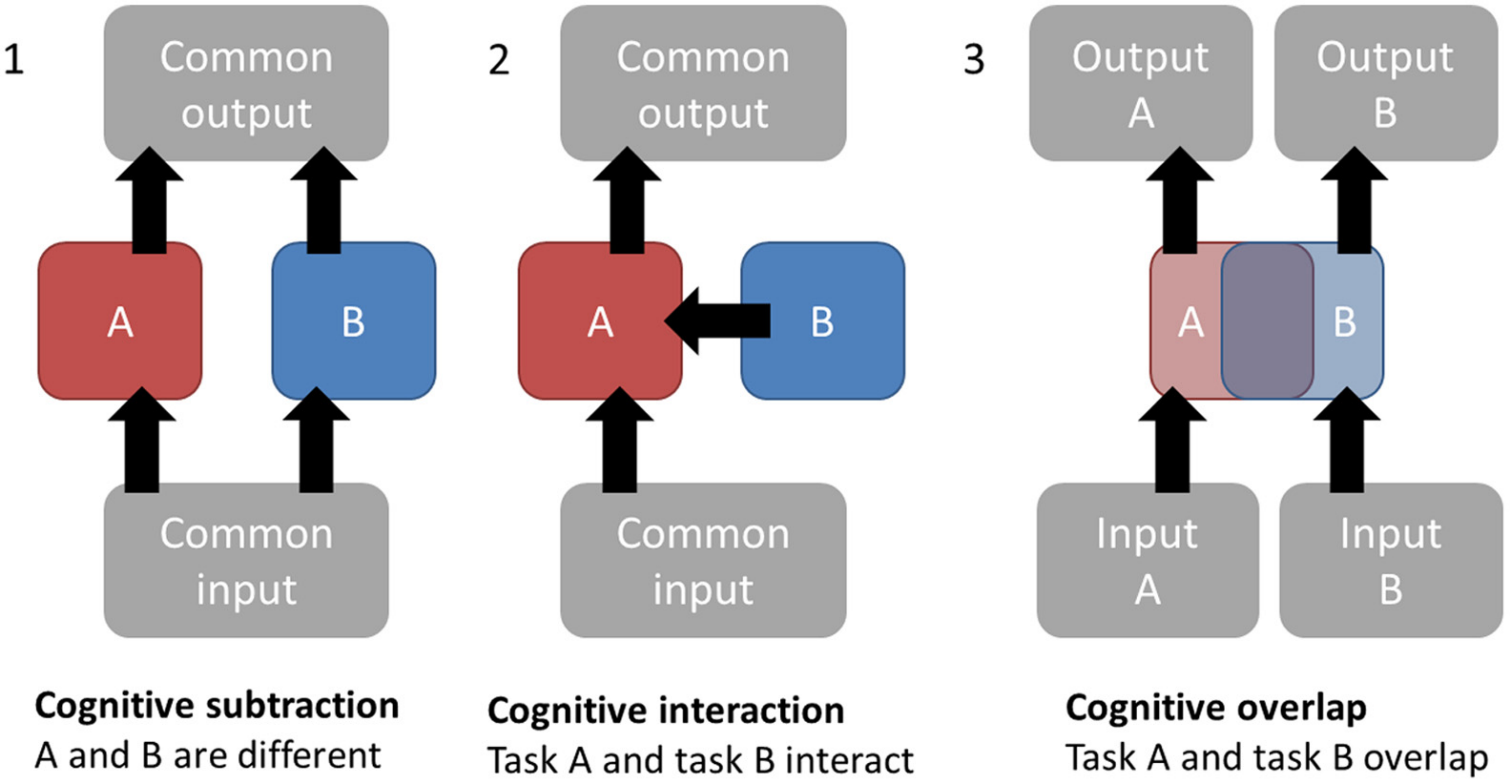
Types of experimental design. Panel 1 shows a cognitive subtraction design where task A and task B engage different central cognitive processes despite having similar inputs and outputs. Panel 2 shows a cognitive interaction where task B influences task A. Panel 3 shows a cognitive overlap where tasks A and B engage similar processes despite having different inputs and outputs.

This classic approach to modeling brain activation with just two distinct experimental conditions is typically called *cognitive subtraction*. The logic of the design, shown in Fig. 2 (panel 1), is that the two experimental conditions both engage similar input systems (for example, visual systems) and similar motor outputs (such as a button press) but differ in the cognitive processing taking place in between. We can then test if brain activity in the condition of interest (condition A) is greater than a control condition (condition B). If a difference between these conditions is found, we can have some confidence that the brain region that is activated is engaged in a process that is specific to condition A. Using a variety of different control conditions can give more confidence about the specificity of an activation pattern, as demonstrated in the classic face-processing paper from Kanwisher et al.^26^

Although cognitive subtraction provides a useful starting point for neuroimaging research, it is also possible to go beyond this. Interaction designs^30^ are one alternative (Fig. 2, panel 2). Here, researchers test if process B modulates or interacts with process A; for example, does the instruction to attend to the left-hand stimulus change the neural activation when participants see images with houses on the left and faces on the right? Panel 3 of Fig. 2 shows a cognitive overlap design, where different input and output modalities are used to test if there are common brain activations for the processing of two distinct types of stimuli. A clear example in fMRI comes from Wicker et al.^31^: participants experienced disgusting or pleasant scents and also, in a separate scan, saw videos of other people smelling disgusting or pleasant scents. A contrast of experienced disgust > experienced pleasant showed activation in the anterior insula that overlapped with the contrast of seen disgust > seen pleasant. This was taken as evidence for common neural processing of disgust stimuli arriving in both olfactory and visual modalities. In all these cases, the brain activation related to the different experimental conditions is being compared with another closely matched experimental condition, not to rest. Alternative approaches include the additive factor method^32^ and methods using repetition suppression,^33^ but a detailed description of these is beyond the scope of the current paper.

#### 2.1.1 Selecting appropriate control conditions

A key consideration in all these types of design is what control condition to use. In general, it is important that the control condition is matched to the experimental condition as closely as possible. For example, in the classic comparisons of brain responses to seeing faces (condition A) or seeing houses (condition B), the images used in the “face” condition and “house” condition should have the same luminance/contrast/colors and the same variety (i.e., 30 different faces compared with 30 different houses, not to 5 different houses). Having good matching between conditions means that the researchers can be confident that any effects are specific to the variable being manipulated and are not driven by low-level visual factors (e.g., luminance/contrast). This is also known as the *fine-cuts* technique,^34^ and using this approach means researchers must pay close attention to what is used as a control condition, just as much as to the experimental condition. This is imperative, as it is only by having a carefully selected contrast between experimental and control tasks that it is possible to draw meaningful conclusions about cognitive processes in the brain.

Building on this fine-cuts idea, we emphasize that good designs typically do *not* need to include a rest condition.^24^ Rest is, in general, a very poor control condition. The human brain is never in a state of rest: a participant is always thinking about something, whether that is their shopping list for dinner, a recent argument with a partner, or just the discomfort caused by the experimental setup. Thus, instructing participants to “rest for 30 s” does not create a brain that is doing nothing, but rather each person will be doing something different with varying cognitive demands, and there is no experimental control over what is happening in the brain.^35^^,^^36^ It is common for people to think about themselves or other people during “rest” conditions; in fact, there is evidence that the “resting-state network” overlaps substantially with brain networks linked to social cognition.^37^ Therefore, “rest” is a particularly poor control condition, especially when a target task is about social cognition or social interaction, because participants are also likely to be engaged in thinking about social topics during rest.

Our argument against “rest” as an experimental condition should not be taken as an argument against studies of the neural “resting-state” when brain activity is measured for several minutes without any experimental task.^38^ Studies of resting-state brain activity may be valuable in many ways, but they are distinct from studies of real-world tasks that are the focus of this paper. A short period of “rest” is also typically included at the start of an fNIRS study to allow participants to settle into the experimental setting, to allow systemic physiology to stabilize, and to establish a baseline reference for quantifying hemodynamic changes using the modified Beer–Lambert law.^39^ Recording brain activity during a short period of rest before the main experiment can assist with the interpretation of task-based fNIRS data due to the association between resting-state and task-induced activation.^40^ However, this is not the same as the use of “rest” as a control condition within a study design. Future studies could explore the relationships between resting-state neural activity, baseline neural activity at the start of a study, and task-related brain activity, but that is a separate question, which is beyond the scope of this paper.

When designing experimental control conditions, the critical idea is that each control condition must be selected in relation to an experimental condition—there is no general “control” that will work across all experimental designs. In a study of visual processing (e.g., seeing faces), the best control condition is likely to also be visual (e.g., seeing houses). In a study of auditory processing, an experimental condition consisting of listening to sentences could be compared with a control condition hearing scrambled or reversed sentences.^41^ In a study of basic motor systems, hand movements can reasonably be compared with rest (no movement), but it might also be useful to compare complex hand movements with simple repetitive hand movements, for example. In a study of social interaction, a suitable control condition might be a demanding nonsocial task such as searching for a letter T among an array of letter Ls^42^ or neatly coloring in a complex form.^43^ In both designing and interpreting the results of neuroimaging studies, it is critical to consider both the experimental and control conditions in relation to each other.

To summarize, experimental design in fNIRS research can learn a lot from the experience of researchers using fMRI (see Table 1 for a summary). We see that block designs are optimal, but event-related designs can also be used with careful consideration of task timing. All these experimental designs are best developed in relation to the specific cognitive processes under investigation and a fine-cuts approach to separating out different cognitive processes in the brain.

**Table 1:** Summary of simple design rules for general fNIRS experiments.

Simple design rules
1. Closely match experimental and control conditions
2. Do not use rest as a control
3. A total of 30 s blocks are optimal
4. Events should be randomized
5. Sample size matters

## 3 How to Take fNIRS into the Real World

One of the major advantages of fNIRS is that participants can move freely and interact with other people, objects, and places in the world. This creates options for a wide variety of experimental designs that may look quite different from the traditional fMRI studies or lab-based cognitive tasks described in the previous section.

One of the first real-world studies conducted using wearable and portable fNIRS is the work by Burgess and colleagues.^15^ This study built upon the need for ill-structured and open-ended situations when investigating prospective memory (PM). Such research examines the challenge that arises when people must remember to do a task in the future (the PM target task) despite various real-world distractions. This mirrors real-world processes, such as remembering to buy milk on the way home from work despite train delays. Laboratory studies cannot easily capture the complexity of such situations; so in this study, Burgess et al. ^15^ used mobile fNIRS to test whether it is possible to assess activation patterns in the prefrontal cortex when people perform a prospective memory task in the real world. The experiment was conducted in the streets of London with no particular preparation of the environment; participants could freely move while wearing a mobile fNIRS device and were asked to find and reach specific PM targets (e.g., a confederate standing on the other side of the street) while also completing ongoing tasks that required attention to the environment (e.g., counting doorbells). Three types of baselines were added to the task design: (1) cognitive baseline (cognitive task without physical activity); (2) walking baseline (physical activity without a cognitive task); (3) environmental baseline (walking around the experimental area). These were included to have periods of time that involved either similar cognitive processes to the experimental PM conditions, and/or a similar level of physical activity, to allow the researchers to disentangle whether significant changes in brain hemodynamics observed during the PM conditions are related to the cognitive demands imposed by the task itself or are a result of systemic artifacts.^7^ The authors compared the social and nonsocial PM conditions against the baselines and found significant activity within the medial prefrontal cortex (PFC) in the PM conditions and higher involvement of the lateral PFC when maintaining social PM intentions compared with nonsocial ones. These results provide evidence of the feasibility of using mobile fNIRS to assess executive functioning outside of the laboratory. It also illustrates the real-world use of a subtraction design with long blocks and a variety of active baseline conditions.

### 3.1 Systemic Physiology

**Fig. 3:**
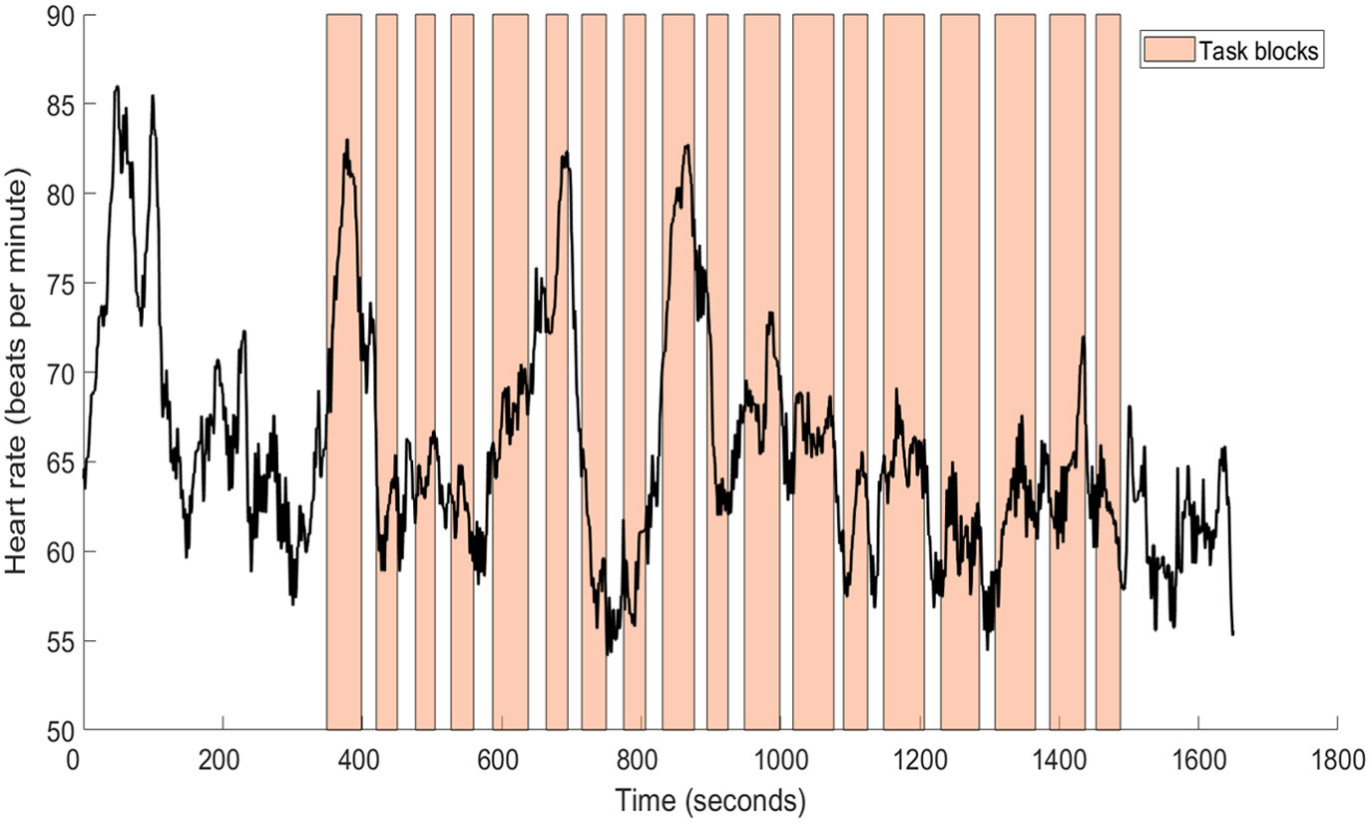
Heart rate can vary drastically even when participants are sitting still while completing simple tasks on a computer—this participant’s heart rate varied from 54 to 86 beats per minute while stationary and completing a simple computer-based math task (which did not lead to a significant increase in stress, as assessed by heart rate variability). These physiological fluctuations could lead to false positives or false negatives if not measured and accounted for in analyses.^7^

An important consideration for real-world fNIRS designs is the interaction of systemic physiological signals with the fNIRS recording. Systemic physiological signals (including heart rate, breathing rate, blood pressure, arterial oxygen saturation, and partial pressure of carbon dioxide) are important because these factors impact the flow of blood to the brain and the oxygenation of blood. Therefore, changes in systemic physiology can change the fNIRS signal even if neural activity does not change, leading to false positives or false negatives.^7^ This has also recently been shown to occur in freely moving toddlers, as young as 3 years of age,^44^ as well as adults. In many cases, this will reduce the signal-to-noise ratio, making it harder to draw meaningful conclusions from the data. However, the issue of systemic physiology is particularly important in cases where physiological changes are related to the tasks that participants engage in (see Fig. 3). For example, if condition A involves participants watching a scary movie where their heart rate and breathing rate both increase, whereas control condition B involves watching neutral movies, then increased brain activity in condition A could be related to the emotion itself or merely to the changes in heart rate and breathing that arise in condition A.

A variety of methods have been proposed to minimize the impact of physiology-related components in fNIRS signals. One option is to regress out superficial contamination via specific short-separation channels that capture superficial blood flow in the scalp, but not brain activity^45^—thus accounting for the potential impact of superficial blood flow changes on the fNIRS data. Another option for reducing the impact of physiology is global mean removal,^46^ which involves calculating the average across all channels (with the idea that this reflects the systemic fluctuations common across the channels that are not directly related to the local brain activity) and then subtracting this average from the data in each individual channel to reduce the impact of widespread physiological changes. One recent study collected fNIRS data from 3- to 7-year-olds, comparing a conventional computer-based task to a dynamic task in an immersive virtual reality (VR) environment, also incorporating short-separation channels. This experiment employed a subtraction design using a go/no-go inhibition task in both standard computerized and immersive VR environments. This VR environment facilitates free participant movement, which is important for ecological validity, particularly in developmental populations. Findings demonstrated that fNIRS data in children as young as 3 years of age can be contaminated by physiological changes (as is the case in adults), and that this can be improved by including short-separation channels in analyses.^44^

An alternative is to take a systemic physiology augmented approach (SPA-fNIRS^47^), enriching fNIRS recordings with concurrent physiological measures (see Yücel et al.^48^ for recommendations on best practices for fNIRS research). Here, physiological signals can be regressed out from the main fNIRS signal. A third option might be to consider physiology at the design stage and ensure that experimental conditions are matched for physiological changes as closely as possible. In the “scary movie” example above, the researchers could include a second control condition that asks participants to breathe fast and perform rapid movements to raise their heart rate a little. Physiological factors should be considered at the experimental design stage, especially in real-world research studies involving participant movement.

## 4 fNIRS for Hyperscanning and Social Interaction

fNIRS data can be collected while two or more people engage in tasks in the same physical space, which enables many new types of research. This technique is known as hyperscanning^20^^,^^49^ and has previously been used for fMRI, EEG, and fNIRS, but fNIRS is the dominant modality.^50^ There are specific challenges involved in designing and interpreting data in hyperscanning studies, and addressing these requires an understanding of both the measures and the theory underlying cross-brain effects. Most hyperscanning studies analyze data using measures that quantify how similar activity is across two brains, also known as interpersonal neural synchrony (INS). Methods to calculate INS include correlations and wavelet coherence measures.^50^ A common finding is that there are similar patterns of activation across the brains of two people engaged in an interactive task.^51^

**Fig. 4:**
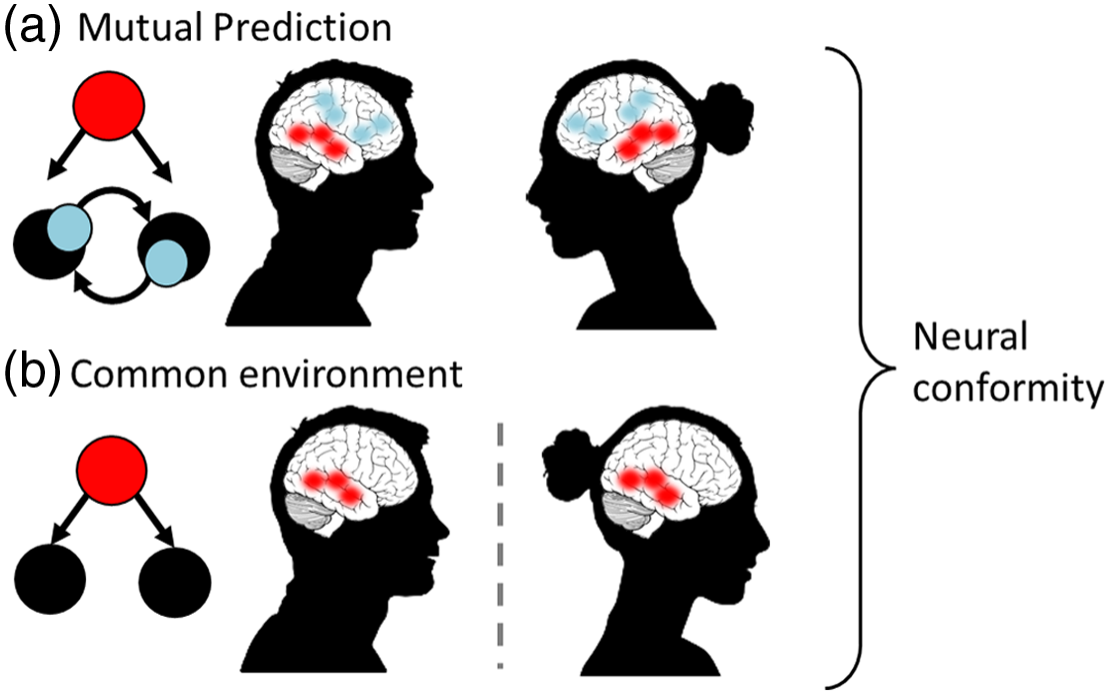
Interpretation of hyperscanning. When two people are engaged in an interaction in the same space (a), they mutually predict one another’s actions (blue). In addition, they may both respond in a similar way to any common inputs from the environment (red). It is also possible to study these responses to common inputs in noninteractive contexts such as sequential fMRI scanning (b). Here, both people experience the same inputs on different days and engage similar brain regions. The similar responses are driven by the common input (red). Analyses that do not separate these two processes can capture measures of neural conformity.

Although there are a variety of interpretations of hyperscanning effects,^52^^–^^54^ one of the clearest is the mutual prediction theory.^55^^,^^56^ This posits that, when two people are engaged in an interaction, each person will both perform actions and predict the actions of their partner [Fig. 4(a)]. Because the two people are in the same physical space, there will almost always be a close correspondence between the performed actions and predicted actions. Because brain systems for performing and predicting actions are overlapping,^57^ there will therefore also be a close correspondence between brain activity patterns in the two people. That is, the two brains will be engaged in mutual prediction^55^^,^^56^ and INS will arise from the prediction-action networks found in each individual. This mutual prediction effect goes beyond just common responses to the same stimulus (Fig. 2, panel 1), because it involves each person actively engaging with and predicting the other.

However, in interpreting hyperscanning effects, it is also important to consider how the two people involved are responding to common inputs from the environment [Fig. 4(b)]. Many studies have used either fMRI or EEG sequential scanning (see Table 2) to capture brain activity from solo participants watching movies and thus experiencing a rich and meaningful audiovisual input.^59^^–^^61^ These studies show inter-subject correlations in brain activity patterns across people. In the context of fNIRS hyperscanning, we distinguish two different types of research questions and thus two different types of experimental design that can be valuable to researchers.

**Table 2:** Underlying mechanisms of inter-brain synchrony: sequential versus hyperscanning.

	Sequential scanning	Hyperscanning
What is brain synchrony believed to reflect (theory and cognitive models)?	Common cognitive processes (common input)	Common cognitive processes (common input) + **interactive** cognitive processes (e.g., mutual prediction)
What is the relationship between the two+ signals?	Entrainment	**Mirroring** *or* **dynamic coupling**^58^
	Movie → A	A ⇔ B
	Movie → B	

### 4.1 Is Neural Conformity Greater Than Chance Level?

One pragmatic option is often to interpret the overall interpersonal neural synchrony level as an index of *neural conformity* that encompasses both mutual prediction and responses to the common environment, and to test if this neural conformity is related to other variables of interest (e.g., learning, affiliation, social relationships). For example, measuring neural conformity in students and teachers during lessons could provide a practical measure of learning or the success of the lesson,^62^ whereas measures of conformity in the context of therapy could be related to the outcomes of the therapy.^63^ Such methods allow us to capture and interpret brain activity in conditions of real-world interactions and relate it to outcomes. In such studies, it is essential to determine if the observed patterns of neural conformity differ from chance.

A common approach to testing if INS is greater than chance is to compare real data with pseudodata. Pseudodata can be created by shuffling recordings between participants (if A and B did a task together, pseudodata might match A to C who did the task on a different day), or by shuffling timings within a recording (match A’s data from minute 0 to 5 of the task with B’s data from minute 5 to 10 of the same session), or by using phase-scrambling^64^^,^^65^ to generate a pseudo-fNIRS signal. In all these cases, the logic is that the pseudodata contains the same core features as the real data, including the experimental context and audio/visual/motor components, but without the precise temporal matching that is present in a live interaction. The comparison between real-dyads and pseudo-dyads, therefore, should reveal brain activity patterns specific to the real-time interactions.^66^^,^^67^ Note, however, that all pseudodata methods that aim to create a “null condition” for brain activity also create a null condition for behavioral coordination and thus do not provide a perfect control for audio/visual/motor influences on the dyad. To understand this, imagine a hyperscanning study in which a person bursts into the lab and shouts at minute 3 of the experiment, prompting a large brain response in both participants A and B that results in a robust INS signal when the two datasets are analyzed together. If a pseudodata analysis is now used to shuffle the brain data of B, using any of the methods above, we would expect the INS linked to the response to the interrupting person (an effect of the common environment) to disappear because the timing of the pseudodata signals is no longer related to the timing of the real-world events in the lab. That is, pseudodata methods do not provide a control condition that can eliminate the effects of a common environment. This means that pseudodata analyses can be a useful way to identify brain regions showing an INS effect, but these analyses do not necessarily reveal the impact of different experimental tasks on INS.

Active control conditions can be a better way to understand the relationship between task and INS effects. For example, Fishburn et al.^68^ tracked the brain patterns emerging between groups of three participants as they completed a puzzle in various conditions. Specifically, INS during joint completion of the puzzle was compared with INS measured while (a) completing the same puzzle alone, (b) watching two other people completing the puzzle, and (c) watching a movie together. The solo task (a) provides a control condition for the motor movements of completing the puzzle, whereas the other two tasks provide controls for visual inputs and co-watching. The use of this active control condition underpins the argument made by Fishburn and colleagues that the greater INS in the joint puzzle condition reflected the coordination or shared intentionality between the participants, and not just the visual or motor aspects of the puzzle-building task. These results are consistent with the claim that there is stronger mutual prediction when people work together on the joint task, driving greater INS. Similar designs have been employed in a variety of contexts and cohorts.^69^^–^^72^

### 4.2 What are the Cognitive Mechanisms Underlying Interpersonal Neural Synchrony?

**Fig. 5:**
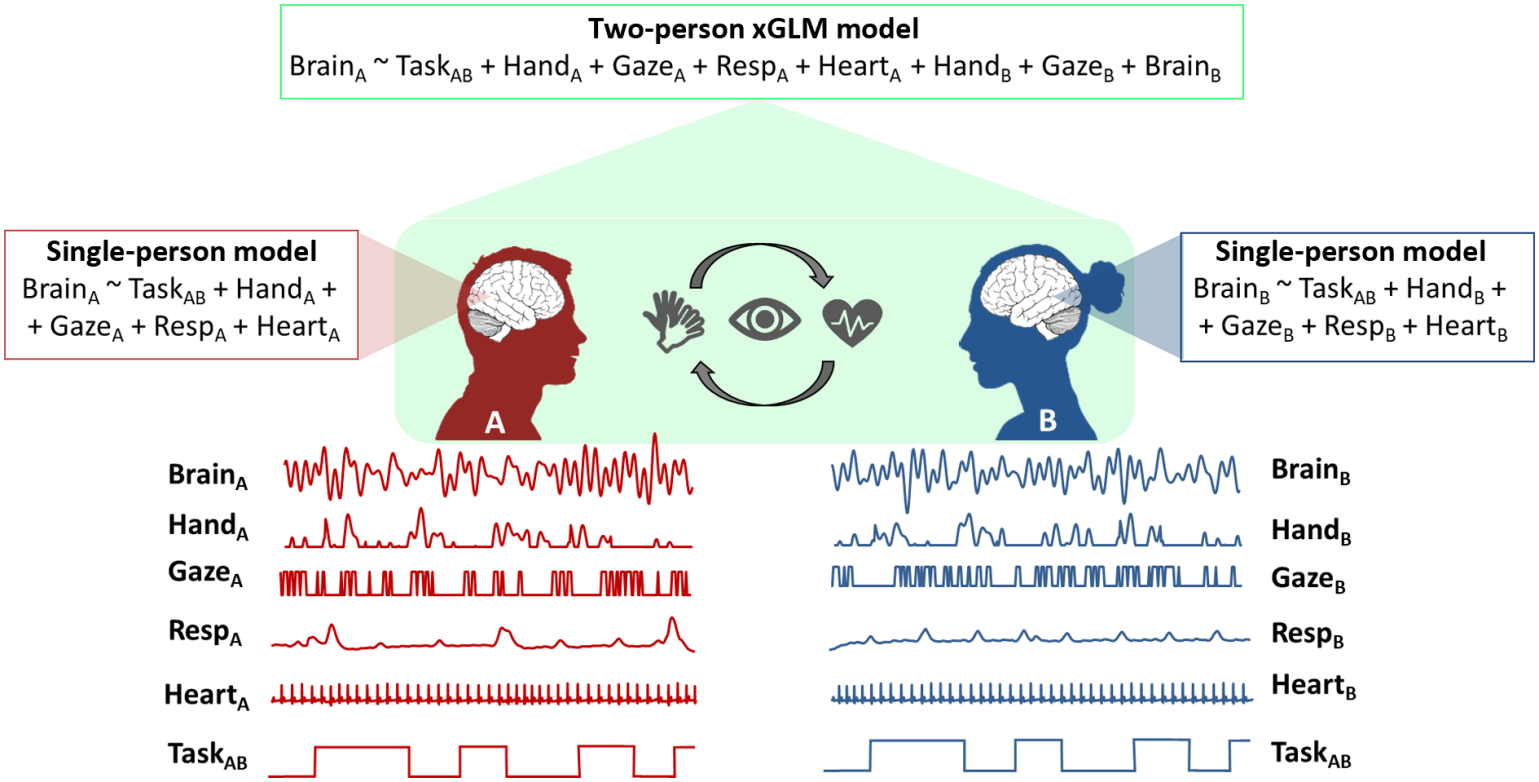
Multimodal data analyses. A real-world fNIRS study can include data from many different modalities, such as brain activity, motion (e.g., hand and gaze), physiology (e.g., respiration and heart), and task blocks. A single-person brain model will test how brain data can be understood as a function of task, motion, and physiological factors. A two-brain xGLM model will add the behavior, physiology, and brain activity of the second person to the model. In both cases, including the behavioral and physiological data enhances the interpretability of the neural models and is much richer than including the task blocks alone.

In the studies reviewed above, INS is taken as a measure of neural conformity, but it is still not easy to distinguish the underlying mechanism that causes the INS effect to arise. Following the mutual prediction theory, coordinated patterns of brain activity arise because behavior is coordinated between the two people involved in an interaction, and, thus, the study of the causes of INS requires detailed recordings and analyses of behavior. The cross-brain GLM approach (xGLM^55^) is one way to incorporate behavioral and physiological signals into models of INS. Here, the multimodal model attempts to explain the single-brain effects as much as possible based on the context and actions of the other participant involved (Fig. 5).

Tracking behavior can be useful to separate out general responses to the environment from specific effects of the social interaction.^53^^,^^55^ A good example of such an approach is the study by Jiang and colleagues,^16^ where the authors recorded brain activity from three interlocutors as they engaged in a group discussion without an a priori leader. The conversation behavior was then coded for each individual within the group to identify the leader’s emergence in terms of quality of communication and frequency of certain behaviors, including initiation and turn-taking. Behavioral variables were used to model brain synchrony, to examine who would synchronize with whom, and when, throughout the discussion. Results revealed a complex dynamic where brain synchrony characterized naturally emerging follower–leader interactions, but not follower–follower interactions, and was associated with moments of “high-quality” communication. Without adopting a multimodal data approach, this INS would have been difficult to interpret.

To summarize, we have identified three major considerations for designing fNIRS hyperscanning studies. First, the appropriate use of pseudodata methods can provide a basic identification of INS. Second, the inclusion of active control conditions gives researchers the ability to separate out different factors and tasks that can increase or decrease INS. Third, detailed recording and integration of behavioral data enables much more informative xGLM analyses that have the potential to identify the single-brain cognitive mechanisms underlying INS. In all these approaches, we suggest it is important to have a good understanding of the underlying theories of INS, to design studies rigorously, and to be cautious in the interpretation of these complex datasets.

## 5 Using fNIRS in Infants and Toddlers

fNIRS is an excellent method for infants and toddlers because it has high tolerance of motion and is very safe. Here, we provide a few examples of the kind of studies that can be conducted, with a focus on different experimental designs.

A classical fNIRS task exploring the development of the social brain tested infants’ responses to seeing a person or object.^28^ This is a block design study alternating between 16 s social trials (i.e., a female actor moving her eyes or mouth, or performing infant-friendly hand games, such as “peek-a-boo”) with 16 s nonsocial baseline trials (i.e., still images of different types of transport, such as cars or helicopters). This simple design is capable of evoking statistically robust activations over the bilateral temporal lobes and has been used on many occasions and in different contexts.^73^^,^^74^

When assessing young infants, the duration of the trial is of particular importance as it is known that the infant HRF peaks slower than the adult one.^75^ Several infant fNIRS studies have adopted the approach taken by Lloyd-Fox et al.^76^ and employed trials lasting 8 to 12 s to maximize the likelihood of allowing sufficient time for the HRF to peak while minimizing the risk of the infant becoming bored or inattentive. The shape and the time-to-peak of the HRF when testing young participants can vary depending on age, state of the participant, and measured brain region. In fact, there are several studies with newborn and young infants that report an inverted HRF (i.e., increasing HbR and decreasing HbO) or noncanonical responses, suggesting that neurovascular coupling might still be immature and may develop during the first years of life.^77^ Therefore, it is important to keep in mind possible differences in the time dynamics of hemodynamic responses in younger populations when designing an fNIRS neurodevelopmental study.

A step toward more naturalistic studies of social neurodevelopment with fNIRS used real people as stimuli, rather than videos or pictures. This was pioneered by a study in which the experimenter talked to 6-month-old infants with direct or averted gaze, and with either infant- or adult-directed speech, showing that these signals modulated infants’ brain activation.^78^ Even with real experimenter–child interactions, this study followed a block design structure, alternating between 15 s trials and 10 s baselines. Given the naturalistic nature of the experiment, with real experimenter–infant interactions, the baseline trials involved the experimenter looking down into a booklet with occasional body movements. This approach was taken to approximately match the degree of movement during the experimental trials while avoiding social engagement with the infant. In these real-interaction studies, the duration of the conditions might slightly vary; therefore, researchers need to consider the implementation of manual event marking or offline coding of the start of the conditions.

Hyperscanning studies have recently expanded into the developmental field. For example, in two studies, mother-infant dyads’ neural synchrony was measured while they engaged in an interactive play session.^79^^,^^80^ Both of these studies found that aspects of the interaction, i.e., turn-taking and affective synchrony, modulated the dyads’ neural synchrony. This approach starts with global measures of the neural conformity between mother and child, but the additional coding of turn-taking behavior and affective synchrony allowed researchers to implement a more detailed analysis relating INS to behavioral synchrony.

**Fig. 6:**
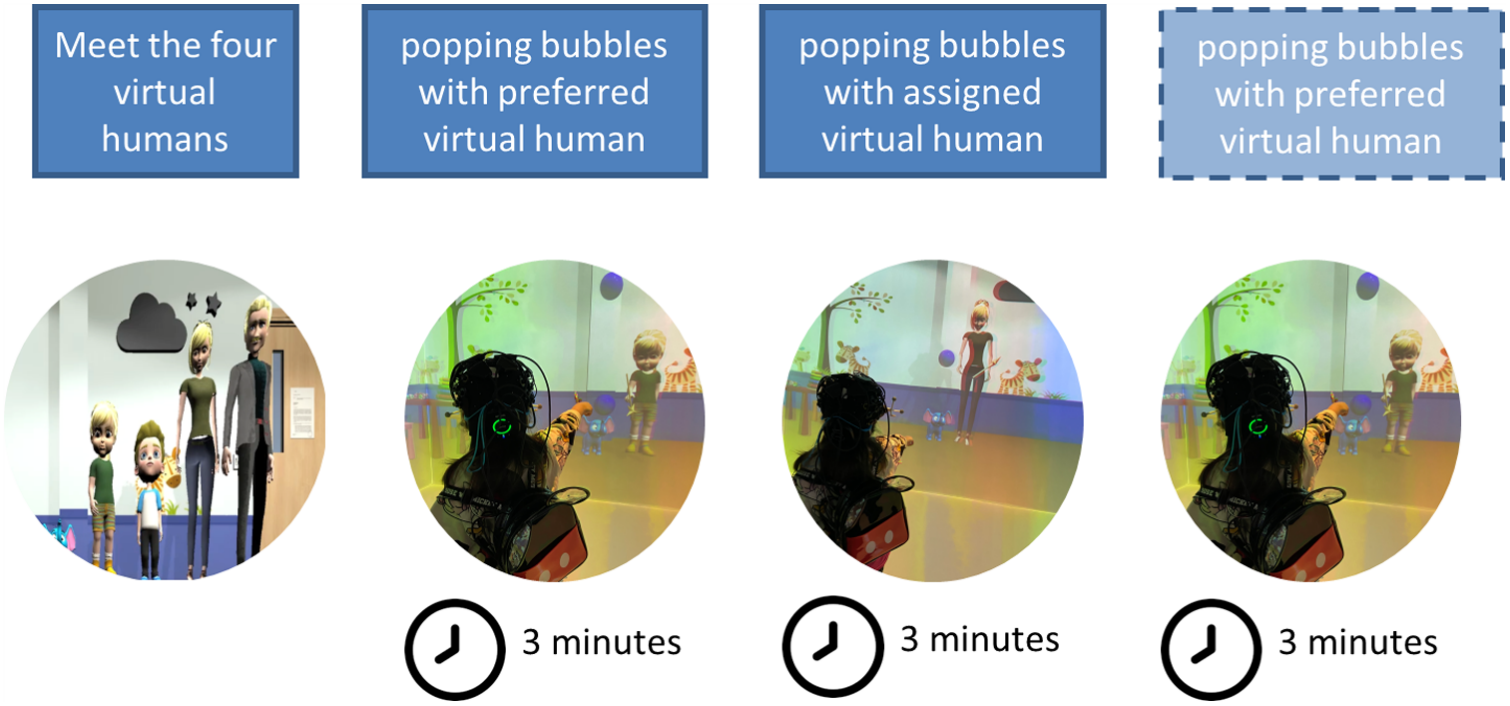
Example of a naturalistic fNIRS task done in a VR setup with 3-to-5-year-olds investigating social preference.^81^ The first part involved participants being familiarized with virtual humans of different ages and genders. Then, the participants were asked to choose a preferred virtual human to play with, popping bubbles for 3 min, and then performed the same task with a randomly assigned virtual human for 3 min. Participants who agreed to keep the equipment on were asked to play again with the preferred virtual human for 3 min.

An exciting frontier for naturalistic social neuroscience studies is virtual reality. Virtual reality environments are the ideal midpoint between the uncontrolled real world and restricted laboratory settings as researchers can control the experimental variables while still immersing participants in a scenario that resembles their real lives. In cave automatic virtual environments (CAVE) systems (but not adult-sized head-mounted VR), wearable fNIRS can be easily implemented to record children’s brain activation as they move and interact with the virtual environment.^81^ In a proof-of-principle study, Bulgarelli et al.^81^ recorded preschoolers’ spontaneous changes in oxygenated and deoxygenated hemoglobin while they interacted with a preferred and a randomly assigned virtual human (Fig. 6).

One interesting methods issue that arises here is the challenge of counterbalancing the order of experimental conditions. This naturalistic study presented conditions in a fixed order to mirror real-life situations, thus precluding counterbalancing. To account for order effects while still preserving the naturalistic aspect of the task, some of the participants were asked to play again with the preferred virtual human at the end of the task (Fig. 6). fNIRS analyses of spontaneous brain fluctuations during the last preferred virtual human condition (instead of the first one), compared with the assigned virtual human, showed similar results as when comparing the original first preferred virtual human condition with the assigned virtual human. This suggests that the different connectivity patterns between the social brain regions found in the two conditions are more likely to be driven by the task itself, and not by order effects. Addressing these considerations during the planning phase of a naturalistic study is important to be able to draw meaningful conclusions and is an example of the novel challenges researchers encounter when designing tasks that emulate real-life interactions while adhering to statistical rigor.^81^

In more naturalistic protocols, having a “rest” block is challenging. As described in Sec. 2.1, “rest” conditions are often a poorly controlled cognitive state where participants are thinking about events outside of the experimental context or may be experiencing boredom or frustration, particularly in the case of young children. As with classic designs described above, an active control condition that matches the experimental condition in terms of movement and visual complexity is much more useful than rest. Virtual reality paradigms lend themselves well to an active control condition, where participants can experience a similar environment with just one factor manipulated, which is a crucial element of a well-controlled experimental design.

## 6 Challenges and Future Directions

The three approaches outlined above—real-world fNIRS, hyperscanning, and developmental research—provide different ways to explore human cognition using fNIRS, and each present unique challenges in designing research studies. In this final section, we consider some issues that can be common across many types of fNIRS studies and discuss how our designs must be adapted to deal with these challenges.

### 6.1 Variability of Real-World Tasks

One of the central features of real-world interactions is their diversity and variability—each conversation with a friend or trip to a new place will involve different topics and scenes and cannot be repeated in exactly the same way by another person, or even by the same person on another day. This means that the real world does not fall neatly into the categories required for an experimental design. Even when participants are given simple repeated tasks that enable data collection (as in many of the examples throughout this paper), the precise timing and behavior may vary from trial to trial and from participant to participant. One option for dealing with this complexity is to record participants’ behavior with a high level of detail and then reconstruct a timeline of the task *post hoc*. Recordings can include motion tracking, video cameras, physiological monitoring, eye-tracking, GPS, and several other measures (see Sec. 6.2 on multimodal imaging). These additional data modalities can be included as additional regressors in GLM analyses to better explain the brain activity patterns of an individual (the single-brain model in Fig. 5). However, coding behavior from video footage is time-consuming and not always accurate. Machine learning and computer vision systems can help,^82^ and useful tools include OpenFace^83^ and DeepLabCut.^84^ These can be enriched using computer vision algorithms that allow the estimation body motion and facial expressions from video recordings.^83^^,^^85^ However, some manual coding is often also required.

In addition to behavior tracking, there is a need for data-driven approaches that are able to accurately recover the timeline of functional events and enable the analysis of fNIRS data recorded in unconstrained settings. The automatic identification of functional events (AIDE) algorithm represents the first attempt to identify the onset of meaningful events directly from the fNIRS data. This method seeks specific patterns in the fNIRS signals (i.e., the shape of the hemodynamic response function) to detect the onsets and offsets of functional brain activity without any *a priori* information of the experimental design.^86^ Burgess et al.^15^ demonstrated the application of AIDE in assessing the neural correlates of prospective memory in the real world (i.e., in the streets of London) and found that the brain activity occurred spatially and temporally in parts of the environment where specific targets of interest were located. In this case, the detected onsets were manually matched to the corresponding behavior from the video recordings. To further improve this and similar algorithms, future work should combine them with behavioral measures and artificial intelligence methods for the automatic classification of events.

### 6.2 Multimodal Imaging

As discussed in Sec. 3, when performing naturalistic experiments, it is often essential to record data in several different modalities (brain, physiology, and behavior) to accurately make sense of the patterns of brain activity measured through neuroimaging devices, in either single-brain or dual-brain models (Fig. 5). From a hardware perspective, fNIRS is particularly suitable for multimodal integration as other devices do not interfere with the optical components,^10^ except in cases where external instruments emit strong infrared radiation (e.g., some motion tracking or eye-tracking systems). The importance of multimodal neuroimaging has been demonstrated in various studies across different applications.

The advantages of having additional signals measured alongside fNIRS are twofold. First, physiological signals are extremely useful in minimizing the systemic interferences impacting the fNIRS signals (i.e., SPA-fNIRS^47^) or to improve the recovery of the hemodynamic response.^87^ These physiological signals can be considered nuisance regressors in methods such as the GLM, and their contribution can be regressed out from the measured fNIRS signals. Second, physiological and behavioral signals can provide a holistic view into the cognitive processes involved and can help in understanding the observed patterns of brain activity, and therefore cannot be considered merely noise. Within the framework of social interactions, for example, previous studies have found that interpersonal synchrony can occur at multiple levels, where interacting people show not only synchronized brain activity but also synchronized heart rate and breathing rate^88^^–^^90^ and synchronized actions,^91^ both during verbal interactions and nonverbal behaviors. This likely happens because any cross-brain synchronization is mediated by the interchange of body actions and signaling such as facial expressions, speech, or eye-gaze. For instance, Greaves et al.^14^ quantified interpersonal synchronization at the brain, physiological, and behavioral levels in pairs of actors and found significant coherence at all three levels in different frequency bands. Therefore, examining interpersonal coordination across modalities can provide key information on what drives brain-to-brain synchrony. The integration of multimodal information in the quantification of interpersonal brain synchrony can be achieved using dual-brain xGLMs,^55^^,^^92^ where the fNIRS signals of one partner can be examined in relation to the brain signals of the other partner, their physiological responses, and behaviors (Fig. 5).

Thanks to the recent hardware developments and sensor miniaturization, we now have the opportunity to simultaneously track the brain, physiology, and behavior in more realistic contexts and in a wider range of applications, populations, and environments. However, multimodal neuroimaging is still not yet routine due to some ongoing challenges. First, fully integrated multimodal platforms are not yet available on the market; the development and use of a minimally obtrusive multimodal setup is still down to the researchers, who have to integrate the sensors themselves, which requires substantial technical skills. Second, data fusion algorithms are yet to be developed that can seamlessly integrate data streams coming from different sources (e.g., brain + behavior + physiology) and automatically combine the information to enable a more thorough interpretation of results.

### 6.3 Statistics and Interpretations

Even when a perfect cognitive experiment has been designed, a researcher must also consider the important issues of statistics and data interpretation. These have been explored in many other papers (referenced below), so here we provide just a short guide to the key issues. Sample size and statistical power are key considerations for all research, but particularly for neuroimaging studies, which are often underpowered. The issue of small sample sizes has been a significant issue for the replicability of neuroimaging research.^93^ A power analysis can be run to estimate the minimum appropriate sample size for a given experiment, and this should ideally be conducted *a priori* to determine required sample sizes for a prospective experiment. Guidance for fMRI research^94^^,^^95^ provides a starting point, but we are not aware of any similar tools specifically for fNIRS research. *Post hoc* power calculations might also be a useful approach.^48^

Large sample sizes are even more critical in the case of research examining individual differences, as data from such studies tends to be highly variable. Lakens and Evers^96^ clearly describe the importance of well-powered research, arguing that low sample sizes can contribute to unreliable results and failures to replicate findings. Moreover, test–retest reliability is critical to any clinical or individual differences approaches. That is, a paradigm that measures the effect of condition A on brain activity in a participant on Monday should be able to measure the same effect size in the same participant on Tuesday if the outcome measure is to be useful as a clinical indicator or diagnostic measure. In studies that aim for clinical use, evaluating test–retest reliability should therefore be an early step in designing any paradigm. A small number of studies have evaluated this using fNIRS, finding moderate to good reliability.^97^^–^^99^ Good experimental designs, precise localization of optodes on the head, and the standardization of protocols and data analysis pipelines may all be important to maximize the reliability of fNIRS.^100^

Obtaining statistically reliable results is important to enable researchers to evaluate the robustness of findings. Many options are available for neuroimaging data, with the fMRI field dominated by parametric approaches.^23^ Similar methods can be applied to EEG data (such as Fieldtrip^101^) and to fNIRS data (such as SPM-fNIRS^102^). These approaches typically model the brain activity at each channel or brain location in each participant with a design matrix and then evaluate statistics at the group level with random field theory providing an appropriate correction for multiple comparisons. Other methods using nonparametric statistics and multivariate statistics are also possible, but some consideration of the issue of multiple comparisons is essential in fNIRS studies to avoid spurious conclusions.

### 6.4 Reverse Inference

Even with a perfect experimental design, there are still some important issues to consider when interpreting fNIRS data. First, there is the challenge of reverse inference,^103^^,^^104^ which has been recognized in fMRI research for decades and similarly applies to fNIRS. Simply put, there is no straightforward mapping between brain activity and cognitive functions. For example, if you show a person in an MRI scanner images of fearful faces, this will reliably activate the amygdala,^105^^,^^106^ and this is a forward-inference (from task to brain). However, if all you know about a participant is that their amygdala is active in condition X, you *cannot* conclude that condition X involves fear faces or the subjective experience of fear. This is because there are many other cognitive processes that also activate the amygdala—learning, surprise, and attention, for example.^107^^,^^108^ The same is true for all other brain regions—many different cognitive tasks activate each region—so we cannot infer a participant’s cognitive state just from knowing brain activation patterns.

This is a challenge because it is very tempting (and would be indeed very useful!) to be able to easily put cognitive labels on brain activation patterns. For example, if we could confidently say that every time the dorsolateral prefrontal cortex (dlPFC) is active, a participant must be working hard on a task and that dlPFC activity is therefore a robust measure of cognitive load, which would be very useful to researchers, engineers, and anyone who wants to use fNIRS in an applied context (for example, for training or interventions). Unfortunately, the issue of reverse inference means that seeing high activity in dlPFC and concluding “this person is working hard” is not valid, and therefore brain activity cannot be used as a direct measure of any one cognitive process.

There are ways to mitigate against this. First, if a study finds an unexpected result of brain activity in area Y specific to a certain task/condition, it is possible to use formal meta-analytic software (e.g., Neurosynth^109^) to obtain a statistic on what other tasks engage the same area. This would help the researcher understand if the task designed might have accidentally involved other cognitive processes or functions not originally intended to be elicited by the task. Second, some argue that within a specific task setting with many conditions designed to precisely isolate a specific process, reverse inference can have predictive power.^110^ Third, empirical studies can test if the mapping between a particular brain region and a cognitive concept is valid under a variety of real-world conditions. For example, if studies show that dlPFC activity really does correlate with cognitive load across a wide range of tasks,^111^ it might be pragmatic to use this as a measure with appropriate caveats. Moreover, although not suited to all experimental paradigms, some methodologies such as transcranial magnetic stimulation and transcranial electrical stimulation can elucidate causal mechanisms of cognitive processes by temporarily altering or disrupting brain activity in a specific region. These methods facilitate direct tests of the impact of disrupting brain activity in a given region on behavior, which can provide causal evidence that complements traditional neuroscientific approaches based on correlational influence.

To conclude, being aware of the problem of reverse inference in neuroimaging research and avoiding lazy assumptions—such as that each locus of brain activity must indicate a particular function or cognitive process—is essential for finding appropriate and valid interpretations of fNIRS data.

## 7 Conclusion

fNIRS is revolutionizing the field of cognitive neuroscience, facilitating the exploration of new research avenues and greatly enhancing naturalistic experiments. In particular, naturalistic studies and developmental studies have benefited due to the portability, robustness to movement and ease of use of fNIRS, and the ability to test awake infants even in resource-limited settings. Nonetheless, it is crucial to carefully design fNIRS studies to ensure that cognitive interpretations and conclusions are valid (Table 3). In this paper, we illustrated how fNIRS researchers can learn from past work in fMRI, and argued that researchers using fNIRS should carefully consider the cognitive mechanisms underlying the patterns of brain activation that can be recorded with our instruments (i.e., cortical structures). We provided a range of examples of how fNIRS can also move beyond traditional fMRI approaches to explore cognition in the real world, in social cognition and in virtual reality. Advances in experimental design in this rapidly developing field are also highly likely, as researchers grapple with the challenges of designing ecologically valid research studies that tap into important aspects of cognition.

**Table 3:** Summary of key design principles.

1. Timing matters: consider hemodynamics when defining trial and event timings.
2. Fine cuts work: compare an experimental condition to an active control to facilitate a strong cognitive interpretation of results. Avoid rest conditions.
3. Behavior matters: fNIRS studies, particularly real-world studies and hyperscanning studies, should record participant behavior and incorporate it into analyses wherever possible.
4. Physiology matters: measuring changes in systemic physiology during a task is important to control for how these measures interact with brain signals, particularly for dynamic tasks.
5. Ecologically valid tasks matter: selecting tasks that engage participants (especially infants and children) and that tap the cognitive processes you are targeting is essential. Tasks that resemble real-world situations may have less experimental control, but they may also evoke more robust and meaningful brain activation patterns than traditional computerized tasks.
6. Statistics matter: issues of experimental power, test–retest reliability, and corrections for multiple comparisons should be considered at the design stage of any study.
7. Cognition matters: to interpret fNIRS results, it is essential to consider how the brain activity patterns relate to different types of information processing and cognitive mechanisms implemented in the brain.

This paper provided a simple primer to help researchers design effective fNIRS studies. Our aim was to summarize current issues and highlight areas where more work is needed. A key challenge for future research is to continue to develop naturalistic fNIRS research that integrates cutting-edge brain imaging methods with real-world, social, and virtual reality scenarios and that can provide new insights into the mechanisms of cognition. We hope this paper will provide a starting point for the next generation of fNIRS researchers to make more use of this important brain imaging tool.

## Data Availability

Data sharing is not applicable to this article as no new data were created or analyzed.
